# An Effective Case for Chyluria by Retroperitoneoscopic Lymphatic Disconnection

**DOI:** 10.1089/cren.2016.0044

**Published:** 2016-04-01

**Authors:** Ikko Tomisaki, Ryoichi Hamasuna, Naohiro Fujimoto

**Affiliations:** Department of Urology, University of Occupational and Environmental Health, Kitakyushu, Japan.

## Abstract

***Background:*** Chyluria is a rare disease in Japan. Lymphatic disconnection is the most effective treatment for patients with Chyluria, and laparoscopic approach is performed as a minimally invasive technique.

***Case Presentation:*** We present a case of a 40-year-old man who referred to our hospital because of recurrence of chyluria. Chyluria had continued for 20 years, and the patient had received retrograde instillations of silver nitrate three times. The patient underwent retroperitoneoscopic nephrolympholysis, and the chyluria disappeared immediately. One year after surgery, chyluria has not recurred.

***Conclusion:*** We treated a patient with chyluria by performing retroperitoneoscopic lymphatic disconnection and this procedure is less invasive and easy to perform.

## Introduction and Background

Chyluria has become a comparatively rare condition in Japan in recent years. We report a case in which we used retroperitoneoscopic lymphatic disconnection to treat a patient with recurrent chyluria who had previously undergone multiple intrarenal silver nitrate injections and obtained good therapeutic results, together with a discussion of the literature.

## Case Presentation

A healthy 40-year-old man visited our hospital as an outpatient with a principal complaint of milky white urine in 1994. Urinary chyle test using the Sudan III staining method was positive, cystoscopy revealed milky white urine draining from the opening of the left ureter, and the left perirenal lymphatic ducts were observed on contrast enhancement, leading to a diagnosis of left chyluria. At first, bed rest and fat-restricted high-protein dietary treatment was provided as conservative treatment, but chyluria did not improve. Then treatment consisted of silver nitrate injection into the left renal pelvis, after which the symptoms improved. The left chyluria recurred in 1998 and 2000; on each occasion silver nitrate was again injected into the left renal pelvis, resulting in an improvement in symptoms in each instance. Milky white urine was observed again in 2014, and the patient was examined in our department once more. He had no history of foreign travel. Physical examination showed no edema of the lower limbs. Urinalysis revealed white and cloudy urinary sediment containing numerous red blood cells/high power field (HPF) and 1 to 4 white blood cells/HPF and proteinuria (5.66 g/day). There were no abnormal findings in peripheral blood, biochemistry, and immunologic tests. No abnormal findings were seen in either kidney or the bladder by ultrasonography. Thoracoabdominal contrast-enhanced CT revealed no tumorous lesions, vascular abnormalities, or lymphatic duct lesions around the kidneys. No abnormal findings were seen in the upper urinary tract by the drip infusion pyelography. Urine cytology showed as benign.

A recurrence of left chyluria was diagnosed on the basis of these findings. As this recurrence followed treatment with three previous silver nitrate injections, we decided to treat it surgically with retroperitoneoscopic lymphatic disconnection.

**FIG. 1.**
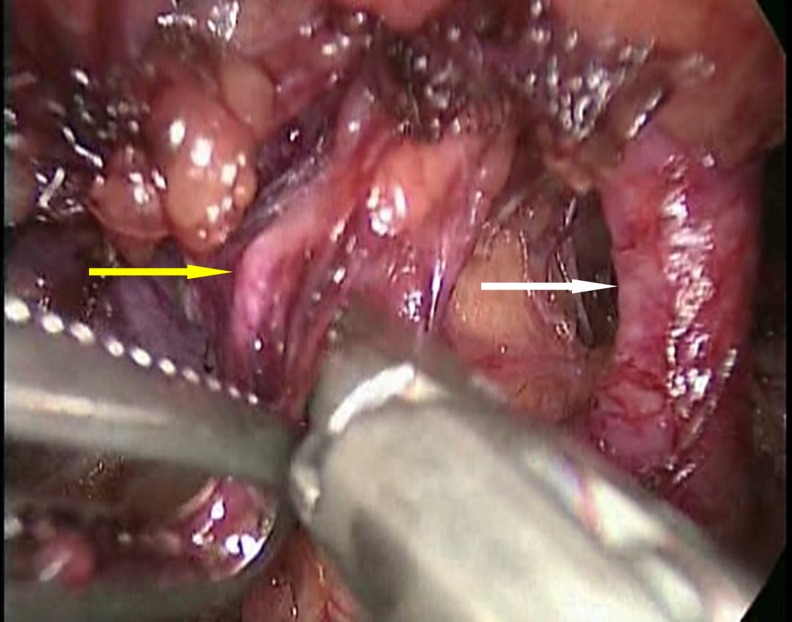
Retroperitoneoscopic view of the dilated lymphatic vessel (*yellow arrow*) and renal artery (*white arrow*).

General anesthesia was induced and the procedure was performed through a retroperitoneal approach. The patient was placed in the right lateral decubitus position, and a 2-cm transverse incision was made on the midaxillary line, 3 cm cranial to the left iliac crest. Once reached, the retroperitoneal space was expanded using balloon dilatation. Then, 12, 11, and 5-mm ports were inserted, and pneumoperitoneum at 8 mm Hg was initiated. Numerous well-developed lymphatic ducts surrounding the renal vessels were evident in the renal hilar region, including some that were dilated in places to a diameter of 2 to 3 mm (Fig. 1). On the ventral side of the renal vein, there was an acinar mass containing a clear yellow liquid, which was considered to be a lymphocele. The lymphatic ducts were mainly transected using a Ligasure^™^ Blunt Tip 37 (Covidien, Tokyo, Japan) so as to leave only the renal artery and vein. The surrounding tissue was also separated from Gerota's fascia around the entire circumference of the kidneys, but no dilated lymphatic ducts were seen, other than those in the renal hilar region. The ureter was also dissected as far as its intersection with the common iliac artery. The operating time was 210 minutes, and the amount of hemorrhage was small.

The milky white urine disappeared immediately after surgery. No lymphatic fistulae were observed and the drain was removed on day 3 after surgery, with the patient discharged on day 8. Outpatient measurements of proteinuria revealed a marked drop to 0.08 g/day, blood tests showed no decline in renal function, and Doppler ultrasonography did not reveal any decrease in renal perfusion. One year postoperatively, there has been no sign of recurrence.

## Discussion

Chyluria can be classified as parasitic or nonparasitic, and filarial infestation is a major cause of parasitic chyluria. With the disappearance of filariasis from Japan, chyluria has become a comparatively rarely encountered condition in recent years. Treatments for chyluria include conservative therapy, which entails rest, dietary therapy with a low-fat diet, and several types of invasive treatments: intrarenal injections, lymphatic duct and lymph node-venous anastomosis, and perirenal lymphatic disconnection. Conservative treatment is mainly used in mild cases, and invasive treatment is indicated in refractory cases or severe cases such as those with clot colic, urinary retention, and trophopathy.

Intrarenal injection of silver nitrate is used in many cases and has a 96.8% success rate, but the recurrence rate is also high at 31.7%, and it takes some time for its effects to appear. The appearance of lower back pain has been reported after intrarenal silver nitrate injection, as have serious complications including renal necrosis and pseudoaneurysm, and it cannot altogether be regarded as a minimally invasive treatment. Lymphatic disconnection, however, has good therapeutic results, with a success rate of 100% and a low recurrence rate of 0% to 3.8%.^1^ The surgical procedure also resembles nephrectomy and is not technically demanding, making this the first-choice invasive treatment for chyluria. The symptoms improve immediately after surgery, making it particularly valuable in severe cases.

Perirenal lymphatic disconnection was formerly performed as open surgery, with endoscopic lymphatic disconnection through the transperitoneal approach first reported in 1995 and through the retroperitoneal approach in 1998.^2,3^ Endoscopic surgery has the advantage of being comparatively less invasive, and its expanded field of view enables reliable lymphatic duct treatment. The retroperitoneal approach provides direct access to the renal vessels and reduces the effect on the peritoneal organs and the risk of damage, making it more suitable for this procedure. The key point in this surgical procedure is to be sure to seal off the intersection between the lymphatic duct and the urinary tract. With respect to the extent of dissection, some reports have described procedures in which it is carried out around the entire circumference of the kidney,^4^ but it is probably sufficient to disconnect the lymphatic ducts in the renal hilar region and around the ureter, where it is possible that a lymphatic duct may drain into the urinary tract.

Reported complications of lymphatic disconnection include mistakenly severing the arterial branch instead of the lymphatic duct, renal artery spasm, and thrombosis.^1^ In the present case, there were locations in which it was difficult to distinguish between the artery and the dilated lymphatic duct. To prevent complications, it is important to use maneuvers that protect the renal artery and to observe the number of vessels and their courses before surgery on thin-slice CT or CT angiography. In terms of lymphatic duct treatment, for fine ducts of a diameter <5 mm, it is sufficient to seal them off with a Ligasure or other vessel-sealing device, but if dilated lymphatic ducts of diameter ≥5 mm are present, then they must be disconnected securely by using a clip or similar device.

## Conclusions

We treated a patient with chyluria by performing retroperitoneoscopic lymphatic disconnection with good results, and this procedure should be considered as a first choice in patients requiring surgical intervention because it is less invasive and easy to perform.

## Abbreviations Used

CT: computed tomography
HPF: high power field
